# Inhibition of hematopoietic prostaglandin D_2_ Synthase (H-PGDS) by an alkaloid extract from *Combretum molle*

**DOI:** 10.1186/1472-6882-14-221

**Published:** 2014-07-05

**Authors:** Rejoice Moyo, Theresa Chimponda, Stanley Mukanganyama

**Affiliations:** 1 School of Pharmacy, College of Health Sciences, University of Zimbabwe, Mt. Pleasant, Harare, Zimbabwe; 2 Department of Biochemistry, University of Zimbabwe, Mt. Pleasant, Harare, Zimbabwe; 3 Biomolecular Interactions Analyses Group, Department of Biochemistry, University of Zimbabwe, P.O. Box MP 167, Mt. Pleasant, Harare, Zimbabwe

**Keywords:** *Combretum molle*, Alkaloid, Hematopoietic prostaglandin D_2_ synthase

## Abstract

**Background:**

Hematopoietic prostaglandin D_2_ synthase (H-PGDS, GST Sigma) is a member of the glutathione S-transferase super family of enzymes that catalyses the conjugation of electrophilic substances with reduced glutathione. The enzyme catalyses the conversion of PGH_2_ to PGD_2_ which mediates inflammatory responses. The inhibition of H-PGDS is of importance in alleviating damage to tissues due to unwarranted synthesis of PGD_2_. *Combretum molle* has been used in African ethno medicinal practices and has been shown to reduce fever and pain. The effect of *C. molle* alkaloid extract on H-PGDS was thus, investigated.

**Methods:**

H-PGDS was expressed in *Escherichia coli* XL1-Blue cells and purified using nickel immobilized metal affinity chromatography. The effect of *C. molle* alkaloid extract on H-PGDS activity was determined with 1-chloro-2, 4-dinitrobenzene (CDNB) as substrate. The effect of *C. molle* alkaloid extract with time on H-PGDS was determined. The mechanism of inhibition was then investigated using CDNB and glutathione (GSH) as substrates.

**Results:**

A specific activity of 24 μmol/mg/min was obtained after H-PGDS had been purified. The alkaloid extract exhibited a 70% inhibition on H-PGDS with an IC_50_ of 13.7 μg/ml. *C. molle* alkaloid extract showed an uncompetitive inhibition of H-PGDS with K_i_ = 41 μg/ml towards GSH, and non-competitive inhibition towards CDNB with K_i_ = 7.7 μg/ml and K_i_^′^ = 9.2 μg/ml.

**Conclusion:**

The data shows that *C. molle* alkaloid extract is a potent inhibitor of H-PGDS. This study thus supports the traditional use of the plant for inflammation.

## Background

The inflammatory process may be defined as a sequence of events that occurs in response to noxious stimuli, an infection or trauma [1]. It is the first response of the immune system to infection or irritation and is a protective attempt by the organism to remove injurious stimuli and initiate the healing process [2]. Inflammation is a self-defense reaction in its first phase and, hence, is regarded as the main therapeutic target and the best choice to treat the disease and alleviate symptoms [3]. Inflammation plays an important role in various diseases, such as rheumatoid arthritis, atherosclerosis and asthma, which all show a high prevalence globally [4].

The inflammatory process is mediated by prostaglandins which are synthesized from arachidonic acid. Prostaglandin D_2_ is an acidic lipid mediator which is responsible for the regulation of body temperature, hormone release, olfactory reaction, sleep, prevention of platelet aggregation and pain responses [5]. PGD_2_ interacts with two types of G protein coupled receptors that is DP_1_ and DP_2_. The DP_2_ receptors are also known as chemo-attractant receptor homologous on T helper 2 cells (CRTH_2_) [2]. DP_1_ receptors are found on murine and dendritic cells and DP_2_ on Th_2_, eosinophils and basophils. DP_2_ receptors mediate eosinophil chemotaxis and are involved in Th_2_ related inflammation [2].

PGD_2_ is formed by the action of two types of prostaglandin D_2_ synthase isoforms that is lipocalin and hematopoietic type [2]. Lipocalin prostaglandin D_2_ synthase is found in the central nervous system, testis and human heart. Production of PGD_2_ is mainly mediated by a glutathione- dependent hematopoietic prostaglandin D_2_ synthase (H-PGDS) [6]. Hematopoietic prostaglandin D_2_ synthase (H-PGDS) is widely distributed in antigen-presenting cells, T helper (Th_2_) lymphocytes, mast cells, and megakaryocytes, where it selectively metabolizes cyclooxygenase-derived PGH_2_ to PGD_2_[7]. H-PGDS is characterised as a member of the Sigma class of GST gene family which catalyses the conjugation of glutathione (GSH) to an electrophilic substrate [8]. The high specificity of the enzyme for the production of PGD_2_ is attributed to the unique structure of the catalytic unit which is deep and wide unlike the catalytic units for other GSTs which are narrow and shallow [5]. Inhibition of H-PGDS has been shown to be very protective in mouse models with allergic airway inflammation [9]. Thus, by analogy H-PGDS appears to be a promising target for the design of anti-allergic and anti-inflammatory drugs.

Non - steroidal anti - inflammatory drugs (NSAIDs) are among the most commonly used drugs worldwide to aid in treating inflammatory conditions. It is estimated that up to 60% of individuals taking NSAIDs will experience side effects and also some of the NSAIDs such as naproxen have been shown to contribute to a 50% higher risk of heart attack and stroke with long term use [10]. Due to these side effects alternative forms of medicine may help in managing inflammatory conditions.

Ethnobotanical knowledge on plants possessing anti-inflammatory and analgesic properties can open up to new drugs in inflammatory disorders [11]. Medicinal plants constitute an effective source of medicines and herbal medicines have been shown to have profound utility with about 80% of rural population depending on it for their primary health care [12]. In Zimbabwe the leaves of *Rhus dentate*, *Ochna pulchra* have been used to treat stomach pains. *Chimbwidi* and *Dalbergia melsnoxylon* have also been used to treat asthma [13].

Ojewole [14], found analgesic, anti-inflammatory and cardiovascular effects of mollic acid glucoside isolated from *C. molle* leaves and antiprotozoal activity from the acetone extract of leaves from the same plant. *C. molle* was found to have anti-asthmatic and anti-tussive activities [15]. In an investigation of the biological activity of different *Combretum* species, *C. molle* was found to have both anti-inflammatory and anti-schistosomal activity [16]. Inflammatory diseases are a major and worldwide problem [17]. An important mediator of inflammation is PGD_2_ which is produced from PGH_2_ by H-PGDS. Very few studies have been done on H-PGDS, which is an enzyme that has been linked to the inflammation process. Of the few studies done it was shown that H-PGDS is associated with inflammation and allergic reactions [5,9].

According to other studies on *Combretum* species, alkaloids have been shown to have anti-inflammatory properties [18]. The effects of un-fractionated alkaloids from *C. molle* were, thus, determined in this study. The main objective of this study was to investigate the effects of alkaloids isolated from *C. molle* on H-PGDS.

## Methods

### Chemicals

Human recombinant H-PGDS was a kind gift from Professor Bengt Mannervik (Uppsala, Sweden). Ethacrynic acid, cibacron blue, CDNB, GSH were products of Sigma Aldrich. All chemicals unless stated otherwise were purchased from Sigma-Aldrich (Steinheim, Germany).

### Plant collection and preparation

The leaves of *C. molle* were collected from Centenary in Mashonaland Central Province of Zimbabwe. The plant was authenticated and classified by Mr. Christopher Chapano, a taxonomist at the National Herbarium and Botanic Gardens (Harare, Zimbabwe). Herbarium plant samples were kept at the Department of Biochemistry, University of Zimbabwe, Harare, Zimbabwe. Leaves of *Combretum molle* were separated from the plant and then dried at an ambient temperature of 50°C in an oven (Memmert, SRG, SchwaBach, , Germany). The dried leaves were ground to a powder using a two speed blender (BL2, ABB, Moulinex, France) so as to optimize the solvent contact during the extraction process. The powders were weighed on a digital balance (Kern EG, Balingen, Germany) and their masses were recorded.

### Extraction

The leaf powder was extracted with ethanol and acetone. An aliquot of 5 g of the powdered sample was weighed on a balance (Kern and Sohn Co., Balingen, Germany) and mixed with 25 ml ammonia and 50 ml 10% ethanol. The mixture was mixed thoroughly on Vortex mixer (Thermolyne Maxi Mix II, IOWA, USA) and placed in a water bath at 40°C for 10 minutes. The mixture was then filtered through a Whatman filter paper 1 and air dried under a fan. The powder obtained was packed in 50 ml test tubes and stored at 25°C for future use.

### Expression and purification of H-PGDS

H-PGDS was expressed from a pJexpress 401plasmid in *E.coli* XL1-blue cells. The gene also coded for hexahistidine tail. Luria Bertani (LB) medium was prepared and kanamycin was added to a final concentration of 50 μg/ml. A volume of 5 ml of the incubated H-PGDS containing *E. coli* cells was added to each of 2 flasks each containing 500 ml media. The expression of H-PGDS was induced by the addition of isopropyl-beta-thiogalactopyranoside (IPTG) after the absorbance (OD) of 0.4, at λ = 600_nm_ was reached and IPTG was added to make a final concentration of 0.2 mM. The cells were then incubated at 160 rpm at 37°C for a further 15 hours in a SI 300 Lab companion, (Jeio Tech, Seoul, Korea). A pellet was obtained after centrifugation at 3 000 rpm for 5 minutes using a Hettich Rotofix 32 A centrifuge (Tuttlingen, Germany). The pellet obtained from centrifuging was lysed with a lysis buffer (pH 8 phosphate buffer 50 mM, 0.3 M sodium chloride, 10 mM imidazole, 1 mg/ml lysozyme).

This mixture was sonicated using a sonicator (Vibra cell, New York, USA) 2 × 20 s treatment stopping at 2 minutes interval to avoid damaging the protein by heating. Phenylmethylsulfonyl fluoride (PMSF) was added to a final concentration of 170 μM to inhibit proteases. This mixture was then centrifuged using a Beckman Optima LE-80 K ultracentrifuge, (Beckman instruments, California, USA) at 105 000 × g for 1 hour. The supernatant was retained while the pellet was discarded. Protein was then purified by nickel immobilized metal affinity chromatography using Ni Cam affinity resin following the manufacturer’s instructions Sigma-Aldrich (Steinheim, Germany). The fractions collected from the column were tested for H-PGDS activity using CDNB as a substrate. The fractions that exhibited activity were pooled and concentrated using an IVSS Vivapore 10 /20 concentrator (VP2001 Satorius Stedim Biotech, Stonehouse, UK) with a molecular weight cut off of 7500 daltons. The concentrated solution was then dialyzed against 2 × 5 L of dialysis buffer (50 mM sodium phosphate pH 8, 1 mM EDTA, 0.2 mM DTT, 0.02% NaN_3_) using a membrane with a molecular cut off of 12 000 daltons.

The purity of the enzyme purification fractions was determined by sodium dodecyl sulphate polyacrylamide gel electrophoresis (SDS-PAGE), carried out on 15% slab gels using a Hoeffer SE Mighty Small II electrophoresis system (Hoeffer Scientific Instruments, California, USA). Protein bands were stained with Coomasie Blue-G.

### Screening for Inhibition of H-PGDS by alkaloids from *C. molle*

The effect of *C. molle* alkaloid extract on H-PGDS was tested at 300 μg/ml. The enzyme activity was determined through the measurement of the conjugation activity with CDNB at 340 nm using SQ Single Beam Scanning UV/Visible Spectrophotometer (United Products and instruments Inc., USA) and was done in quadruplicate. For the determination of IC_50_, a 2 fold serial dilution of *C. molle* extracts were carried out from 0 to 300 μg/ml in a 96 well plate. The assay with CDNB was adapted for measurement of absorbance with a SpectraMax Plus microplate spectrophotometer equipped with a kinetics mode (Molecular Devices, Sunnyvale, California, USA) at 380 nm using an extinction coefficient of 7.825 μM^-1^ cm^-1^.

### Determination of time-dependent effects

The incubation mixtures contained H-PGDS (final concentration 0.0625 μM), 0.2 M potassium phosphate buffer pH 7.4 with 0.2 mM EDTA, and (15, 30, 60 μg/ml) *C. molle* alkaloid extract. The incubations were carried out at a temperature of 30°C. The experiment was carried out at timed intervals from 0–1 hour, beginning with incubation for the 1 hour sample and lastly with the 0 hour time sample. Immediately, 20 μl of each sample was withdrawn and assayed for GST activity. These incubations were run in parallel with positive and negative controls. The negative control contained H-PGDS and buffer. Cibacron blue was used as the positive control.

### Determination of kinetic constants for H-PGDS using CDNB and GSH as substrates

The effects of alkaloids from *C. molle* on the kinetics of H-PGDS were determined as described previously [19]. Activity with CDNB was measured by determining the absorbance with a SpectraMax Plus microplate spectrophotometer equipped with a kinetics mode (Molecular Devices, Sunnyvale, CA, USA). The K_*m(app)*_ and V_*max(app)*_ were determined using GraphPad Prism™ version 5.00. The K_*i*_ values with respect to GSH and CDNB, as well as the type of inhibition were determined. The type of inhibition was deduced by determination of trends of K_*m*_ and V_*max*_ values with increase in natural product concentration. To determine the trend, the means of the K_*m*_ (or V_*max*_) values with increase in inhibitor concentration were compared by performing a one-way ANOVA with Dunnett’s post test using Graph Pad 5.00 (Graph Pad Prism Inc. San Diego, CA, USA). The inhibition constant, K_*i*_, was determined by means of re-plots [19]. The type of re-plot depends on the type of inhibition, for example, plotting 1/V_*max*_ versus inhibitor concentration for non-competitive inhibition will give K_*i*_ as the intercept on the baseline.

### Statistical analysis

Data analyses were performed using GraphPad Instat software® (GraphPad Prism Inc. San Diego, CA, USA). Levels of significance were determined using ANOVA using the Dunnett post test were all columns of treatments were compared to the control. All data were expressed as mean ± standard deviation. P ≤ 0.05 values or less were considered to indicate statistically significant difference).

## Results

### Coomassie blue staining and Western blot for poly-His tag

H-PGDS was expressed in *E. coli* and was purified by Nickel affinity chromatography. The H-PGDS was purified to homogeneity and a single band was obtained on SDS-PAGE analyses. The molecular weight of the protein was 23.4 kDa and the specific activity was 24 units/mg.

### The effects of *C. molle* alkaloids on H-PGDS activity

**Figure 1:**
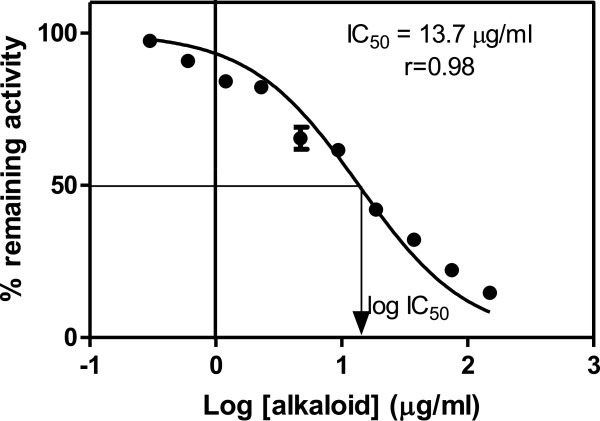
**The inhibitory effect of *C. molle *on GST Sigma with increasing concentration of plant extract.** The concentration of natural product required to bring about 50% inhibition of GST activity, the IC_50_ value, was determined by plotting sigmoidal dose response curves of percent enzyme activity *vs.* log natural product concentration using GraphPad Prism version 5.00 (GraphPad™ Software Inc., San Diego, California, USA). The IC_50_ value determined from the graph was 13.7 μg/ml.

The effects of the alkaloids from *C. molle* on the activity of H-PGDS activity were assessed using the CDNB assay for GSTs. Inhibition activity of the extract at 300 μg/ml plant concentration was determined as the percentage remaining activity of the enzyme in the presence of the plant extract. The percentage inhibition obtained was 70%. The IC_50_ for the alkaloids was determined spectrophotometrically using CDNB as the substrate. Figure 1 shows the sigmoidal dose response curve for the determination of the IC_50_ for the alkaloids which was found to be 13.7 μg/ml.

### Time-dependent effects of alkaloids on H-PGDS

**Figure 2:**
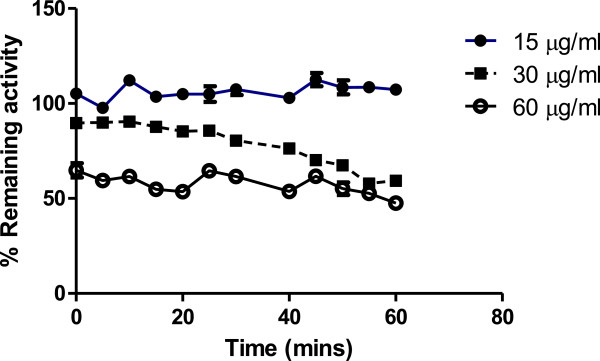
**The time-dependent effects of the alkaloids on GST Sigma over 60 minutes at concentrations 15, 30 and 60 μg/ml of plant extract.** The incubations of the enzyme and the plant extract were carried out at a temperature of 30°C for a period of 0–1 hour. Samples of 20 μl of each sample were withdrawn at timed intervals and assayed for GST activity. The H-PGDS was not inactivated, however, there was a dose-dependent decrease in enzyme activity with time.

To assess if the alkaloid extract could inactivate H-PGDS, the time-dependent effects of the enzyme by *C. molle* alkaloid extract were carried out over an hour and the values obtained were analyzed and presented graphically as % remaining activity against time as shown in Figure 2. The figure shows results for the effects of 15 μg/ml plant extract (low concentration), 30 μg/ml plant extract (middle concentration) and 60 μg/ml plant extract (high concentration). The results show that the effects of the alkaloids were direct, concentration-dependent and did not depend on time. Cibacron blue was used as the positive control.

### The effects of the alkaloids on H-PGDS kinetics

**Figure 3:**
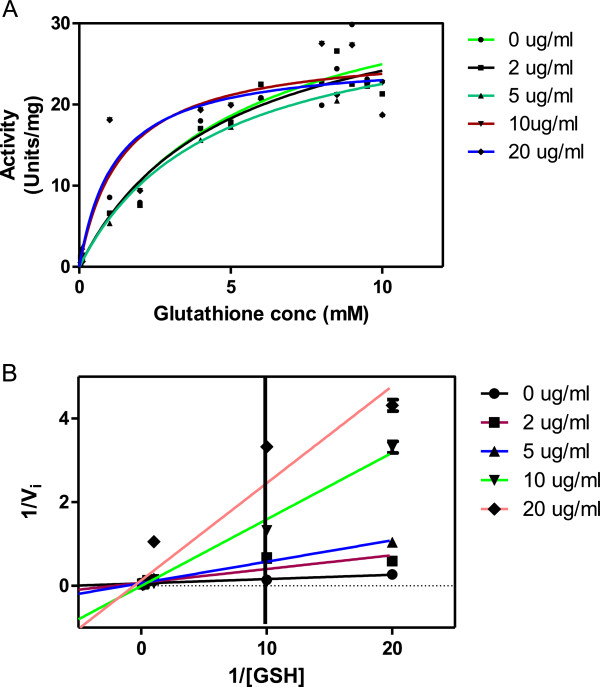
**Inhibition profile of *C. molle *alkaloids on H-PGDS. A** shows the Michaelis-Menten plot and **B** the Lineweaver-Burk plot of GST Sigma. In the assay H-PGDS activity was measured with varying concentrations of GSH and different inhibitor concentrations; 0, 2, 5, 10 and 20 μg/ml whilst CDNB concentration was fixed at 1.5 mM.

**Figure 4:**
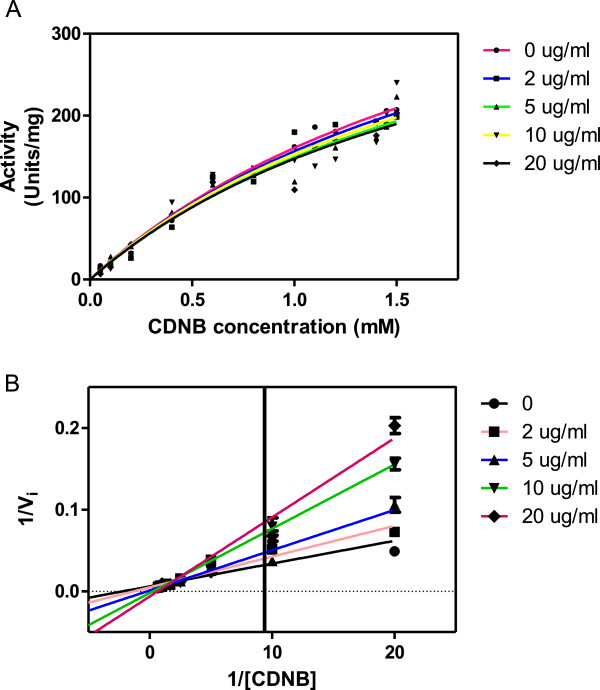
**Inhibition profile of *C. molle *alkaloids on GST Sigma. A** shows the Michaelis-Menten plot and **B** the Lineweaver-Burk plot of GST Sigma. In the assay GST Sigma activity was measured with varying concentrations of GSH and different inhibitor concentrations; 0, 2, 5, 10 and 20 μg/ml whilst GSH concentration was fixed at 5 mM.

**Figure 5:**
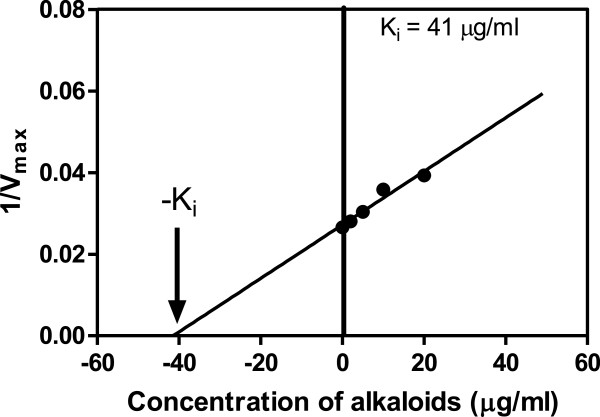
**Secondary plot to determine K**_**i **_**for the alkaloids.** Based on the type of inhibition, a re-plot of 1/V_max_ versus [I] was used to determine K_i_^GSH^ values of alkaloids, since an uncompetitive type inhibition for GST Sigma with respect to GSH was obtained.

**Figure 6:**
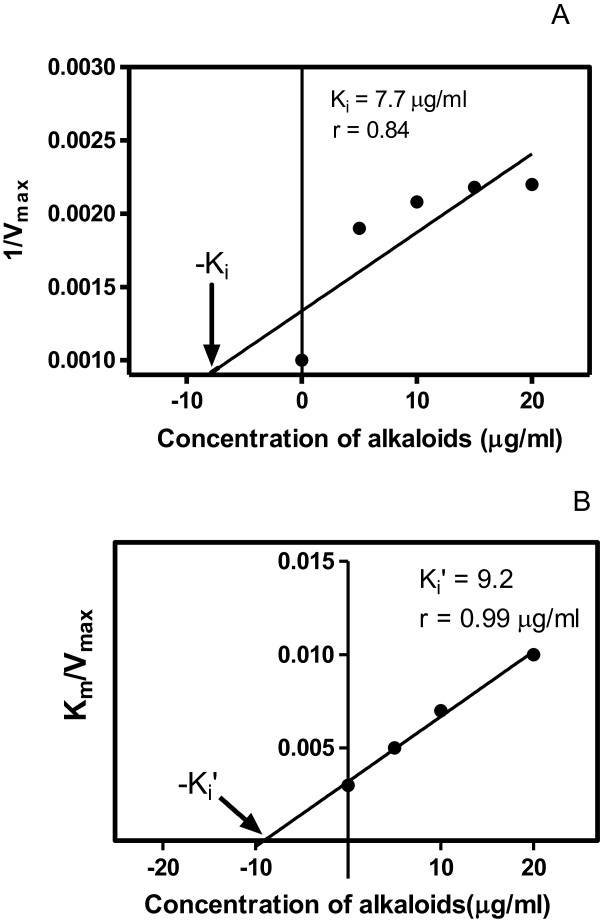
**Secondary plots to determine K**_**i **_**for the alkaloids for GST Sigma with CDNB as the substrate. A** Non-competitive inhibition was obtained with respect to CDNB, and a re-plot of 1/V_max_ versus [I] was used to determine K_i_^CDNB^. In **B**, a replot of K_m_/V_max_ versus [I] was used to determine K_i_′^CDNB^ for the alkaloids. The X- intercept as indicated by the arrow gave the value -K_i_ which was used to calculate the K_i_ and K΄_i_ respectively. These gave values of K_i_ and K_i_′ of 7.7 and 9.2 μg/ml. Correlation coefficients of 0.84 and 0.99 were obtained for plots **A** and **B** respectively.

Based on the results for the inhibitory effects of alkaloids, their kinetics with H-PGDS were determined. The trend in changes of K_*m*_^GSH/CDNB^ and V_*max*_^GSH/CDNB^ values with increase in alkaloid concentration was used to determine the type of inhibition [20]. The predominant type of inhibitions with respect to the G site (GSH) was uncompetitive and with the H site (CDNB) was non-competitive. Figures 3 and 4 shows the Michaelis-Menten and Lineweaver-Burk plots with GSH and CDNB as these substrates were varied respectively. Figures 5 and 6 shows the secondary plots for determination of K_i_ and K_i_′ values for the alkaloids with GSH and CDNB respectively on H-PGDS. The data for the effects of the alkaloids on H-PGDS kinetics was summarised in Table 1. It was shown that the K_cat_/K_m_ for GSH was increased with an increase in the inhibitor concentration and that the K_cat_ for both substrates decreased with an increase in inhibitor concentration.

**Table 1:** **The effects of alkaloids from *C. molle *** **on the kinetic properties of GST Sigma**

**[ *C.molle *]**	**K**_**cat**_**(S**^**-1**^**)**	**K**_**cat**_**(S**^**-1**^**)**	**K**_**m(**_**mM)**	**K**_**m**_**(mM)**	**K**_**cat**_**/K**_**m **_**(S**^**-1**^ **mM**^**-1**^**)**	**K**_**cat**_**/K**_**m **_**(S**^**-1**^ **mM**^**-1**^**)**
**(μg/ml)**	**GSH**	**CDNB**	**GSH**	**CDNB**	**GSH**	**CDNB**
0	171.2 ± 1.7	2366.8 ± 38.6	5.2 ± 0.04	2.25 ± 0.04	33.17 ± 0.06	1051.2 ± 1.03
2	162 ± 0.3	2281.3 ± 3.5	4.82 ± 0.12	2.00 ± 0.02	33.62 ± 0,80	1136.14 ± 6.70
5	149.5 ± 2.6	2218.6 ± 37.3	3.41 ± 0.007*	2.17 ± 0.007	43.82 ± 0.86	1020.19 ± 13.83
10	127.1 ± 5.3*	2118.4 ± 31.2	1.40 ± 0.02**	2.06 ± 0.02**	90.92 ± 4.81	1028.83 ± 5.96
20	115.6 ± 1.9***	2074.5 ± 3.2*	1.14 ± 0.06**	2.02 ± 0.02**	101.9 ± 3.94	1029.36 ± 10.33

One-way analysis of variance test (ANOVA) with Dunnett’s Multiple Comparison Test was used to analyse the results. Values represent the mean ± SD for N = 2. The values with a p-value < 0.05 or less were considered statistically significant. *P < 0.05, **P < 0.01 , ***P < 0.001. Graphical and Statistical analyses were carried out using Graphpad Prism 5® Software (Version 5.0, Graph pad Software Inc, San Diego, USA).

## Discussion

Inflammation is a major worldwide health burden in both developing and developed countries [17]. Chronic inflammation can lead to various conditions such as cancer, asthma, rheumatoid arthritis, atherosclerosis, periodontitis, and hay fever [3]. The search for safe and efficacious agents for use in inflammatory conditions is ongoing with the objective to find new drugs which are efficacious but with few side effects. Conventional medicines have been shown to possess many side effects and, thus, effort is being put into research or new drugs [21]. An important prostaglandin involved in inflammation is PGD_2_ which is produced by H-PGDS. H-PGDS negative mice generated by standard gene targeting technology showed diminished symptoms of disease indicating diminished inflammatory reaction in the absence of H-PGDS [22].

A considerable number of studies have suggested that extracts or active principles obtained from *Combretum* species have a broad spectrum of biological activities, including antibacterial, antiprotozoal, anticancer, cytotoxic, analgesic, anti-inflammatory, hepatoprotective and antiviral activities [23]. *C. molle* is used in traditional medical practices in Zimbabwe to treat pain and inflammation [24]. The aim of the present study was to collect information on the possible pharmacological and molecular basis for the efficacy of the plant alkaloids on the effective management of inflammation. The preliminary studies in our research group showed that the crude plant leaf extract of *C. molle* brought about 87% inhibition of the H-PGDS. An alkaloid extract from *C. molle* was then assayed for its activity against H-PGDS.

Alkaloids are abundant in the leaves of *C. molle* and have been reported to have significant pharmacological activities. Previous studies on this plant led to the isolation of triterpenoids glycosides, tannins, alkaloids, saponins, stilbenes, triterpene saponin oleanone trypetitepene, arjunolic acid and mollic acid glucosides which demonstrated cytotoxic, antifungal, antimicrobial and anti-inflammatory activity [25]. According to the histochemical studies done in a previous study [26], the main constituents of *C. molle* leaves were found to be phenolics, flavonoids and alkaloids.

Analgesic and anti-inflammatory properties of mollic acid glucoside (MAG), an alkaloid, a 1α-hydroxycycloartenoid extracted from *Combretum molle* leaves have been investigated in mice and rats [25]. The results of the laboratory animal study indicate that MAG possesses analgesic and anti-inflammatory effects in the mammalian models used. The author suggested that MAG possesses both centrally- and peripherally-mediated analgesic effects [27]. In an investigation of the biological activity of different *Combretum spp*[16], *C. molle* was found to have both anti-inflammatory and antischistosomal activity. These findings may explain the traditional use of the plant against malaria and pain. This study also contributes to the validation of the popular use of this plant species in the treatment of inflammation.

H-PGDS is specific for and selectively and effectively isomerizes PGH_2_ to PGD_2_, thus, efforts are being put into searching for potential HPGDS inhibitors [5]. The activities of other GSTs have been reported to be inhibited by S-hexyl glutathione (GSH) and its conjugation with 1-chloro- 2, 4-dinitrobenzene (CDNB) [28]. In the present investigation, *C. molle* alkaloids were tested for GST inhibition *in vitro.* The effect of *C. molle* alkaloids on H-PGDS was tested at 300 μg/ml concentration of the plant extract. The inhibition profile was concentration-dependent (Figure 4). Other data also showed concentration-dependent inhibition of cytosolic GSTs when *Mitragyna speciosa* extract was added into the reaction mixture [29]. In that study, the methanolic extract showed the highest GSTs specific activity inhibition (61%), followed by aqueous (50%) and total alkaloid extract (43%), respectively [29]. In this study, *C. molle* alkaloids reduced the enzyme activity by 70%, thus, showing that the fractions were potent inhibitors of H-PGDS. Compounds that inhibit GSTs can prove to be potent drugs [30]. Since the whole leaf extract from *C. molle* was shown to be a potent inhibitor for H-PGDS, further studies to determine the IC_50_ values for alkaloids were carried out. *C. molle* alkaloid extract exhibited inhibitory effects on H-PGDS with an IC_50_ of 13.7 μg/ml. Since the IC_50_ value for the whole leaf extract was found to be 16.7 μg/ml, it suggests that the inhibitory effects in the leaf extract were mainly due to the presence of alkaloids in *C. molle*.

However, the IC_50_ value for the alkaloids was high as compared to other GST inhibitors namely, hematin (3.16 μg/ml), tributyltin bromide (2.2 μg/ml) and for S-hexylglutathione (7.8 μg/ml) [31]. The difference can be ascribed to the fact that this was a mixture and not pure compounds. In a previous study [32], it was found that the pure plant natural products ellagic acid and curcumin were potent inhibitors of GSTs with IC_50_ values of 0.6 and 0.9 μg/ml respectively and, therefore, purified phytochemicals maybe be more effective than mixtures.

To determine if alkaloids from *C. molle* had other modes of inhibition of H-PGDS, time-dependent incubations of the alkaloids with the enzyme were carried out. *C. molle* alkaloids failed to inactivate H-PGDS (Figure 4). Activity at the beginning of the reaction was concentration-dependent and lower than that of cibacron blue at all concentrations. Thereafter, with progression in time, the activity remained constant showing that the effects of the alkaloids were not time-dependent but were reversible.

It was shown that the plant extract exhibited an uncompetitive type of inhibition with regards to GSH as both K_m_ and V_max_ were decreasing and produced a K_i_ of 41 μg/ml. Thus, the extract binds to the enzyme – substrate complex only [33]. The inhibition was non-competitive with respect to CDNB characterised by K_i_ value of 7.67 μg/ml and K_i_^′^ of 9.18 μg/ml. This suggests that the extract binds to the free enzyme and to the enzyme – substrate complex [33]. In non-competitive inhibition, substrate can still bind to the enzyme-inhibitor complex. However, the enzyme-inhibitor-substrate complex does not proceed to form product and the value of V_max_ decreases to a new value while the value of K_m_ is unchanged [33]. In a previous study, the K_i_ values for GSTs were 84.132 and 180 μg/ml respectively for *T. diversifolia, C. rotundus* and *H. suavolens* extracts and, hence, *C. molle* alkaloid extract with lower kinetic constants was a more potent GST inhibitor [34].

Although several studies have investigated the role of PGD_2_ in inflammation, the role of PGD_2_ in the host immune response has been scantly studied [35]. Inflammation in H-PGDS knockout mice was found to be more severe during the onset phase arising from a cytokine imbalance which resulted in enhanced polymorphonuclear leukocyte and monocyte trafficking [36]. Prevention of H-PGDS activity specifically either through gene knockout leads to impaired clearance of lymphocytes and macrophages from sites of inflammation [36]. This may be the case when H-PGDS is inhibited by *C. molle* alkaloid extract. H-PGDS contributes to the production of the D and J series of prostanoids in the immune system and is involved in allergic inflammatory response. Since H-PGDS is present in mast cells, Th2 cells, and other leukocytes, it is thought to be mainly responsible for PGD_2_ production during allergic responses. Inhibition of H-PGDS will reduce the production of PGD_2_ and, hence, result in a decrease in allergies [35].

The substrate used in this study was CDNB which is not the physiological substrate for H-PGDS. However, *C. molle* alkaloid extract is more likely to possess anti-H-PGDS activity even in the presence of PGH_2_ the physiological substrate. The K_m_ of H-PGDS obtained for PGH_2_ was 0.2 mM [37] and in this study we obtained a K_m_ of 2.25 mM for CDNB. Although these values are within a magnitude of difference, *C. molle* alkaloid extract is still likely to have some inhibitory effects on H-PGDS in the presence of PGH_2_. Further experiments using PGH_2_ as a substrate are needed to verify this claim.

## Conclusion

In conclusion, alkaloids from *C. molle* were shown to have inhibitory effects on H-PGDS. The inhibitory effects were lower as compared to cibacron blue, a standard H-PGDS inhibitor. The alkaloids exhibited non-competitive and un-competitive inhibition of H-PGDS with respect to CDNB and GSH respectively. However, potent activity *in vitro* cannot be directly correlated to potent activity *in vivo* due to other factors such as metabolism within cells and the presence of other compounds in the cell which may prevent binding of the required compound to its target site. The effect of *C. molle* alkaloid extract on H-PGDS using PGH_2_ needs to be determined. This study has, therefore, identified alkaloids from *Combretum molle* with potential anti-inflammatory activity and may, therefore, serve as sources of lead compounds for anti-inflammatory drug development. The inhibitory effect of alkaloids from *C. molle* also validates the use of its extracts in traditional medicines to reduce inflammation.

## Competing interests

The authors report no conflicts of interest. The authors alone are responsible for the content and writing of the paper.

## Authors’ contributions

RM conducted all the experimental studies and data analysis with the assistance of TC. SM conceptualised and designed the study. TC and SM finalized the manuscript. All authors read and approved the final manuscript.

## Pre-publication history

The pre-publication history for this paper can be accessed here:

http://www.biomedcentral.com/1472-6882/14/221/prepub
